# The Art of Endowment

**Published:** 1918-11-16

**Authors:** 


### The Art of Endowment.

A FUNCTION AT BARNSLEY.

IN the Fitzwilliam Ward of the Beckett Hospital, Barnsley, a pleasant ceremony was lately held, when a third bed in the ward was endowed in the presence of a representative gathering. The donors were the shareholders of the Barnsley Brewery Company, who gave £1,000 in National War Bonds. Mr. C. J. Tyas, J.P., introduced the chairman of the brewery company, and asked him to make the presentation. Mr. Lucas then formally handed to the senior trustee of the institution, Mr. E. G. Lancaster, the £1,000 War Bonds. Mr. Lancaster, in gratefully accepting the gift, assured the donors that it was all the more acceptable because it came at a hard time. He moved a hearty vote of thanks to Mr. Lucas, to Mr. Wells, who was the chief mover in the presentation, and to the shareholders for their generosity.

Mr. Alfred Whitham, chairman of the committee, in seconding, said a deep debt of gratitude was due to the present staff of honorary medical officers for their excellent work. The nursing staff was second to none, and in Miss Burkhill they had an experienced matron, whose services they hoped to retain for many years. Sir Joseph Walton, M.P., who supported, said that the example of the Barnsley Brewery Company's shareholders was worthy of imitation. His own admiration of hospitals and nurses had been enhanced by four visits, each of a few weeks' duration, to France during the war. The work that was being done there in the interests of suffering humanity was magnificent. One good result which had come out of the war was improved methods of surgical treatment. The resolution having been carried, Mr. Wells briefly responded, and hoped that ere long more beds would be similarly endowed.

